# Light potentials of photosynthetic energy storage in the field: what limits the ability to use or dissipate rapidly increased light energy?

**DOI:** 10.1098/rsos.211102

**Published:** 2021-12-15

**Authors:** Atsuko Kanazawa, Abhijnan Chattopadhyay, Sebastian Kuhlgert, Hainite Tuitupou, Tapabrata Maiti, David M. Kramer

**Affiliations:** ^1^ MSU-DOE Plant Research Lab, Michigan State University, East Lansing, MI 48824, USA; ^2^ Department of Chemistry, Michigan State University, East Lansing, MI 48824, USA; ^3^ Department of Statistics and Probability, Michigan State University, East Lansing, MI 48824, USA; ^4^ Biochemistry and Molecular Biology, Michigan State University, East Lansing, MI 48824, USA

**Keywords:** non-photochemical quenching, photodamage, proton motive force, qE, photosynthetic control fund, unsupervised learning

## Abstract

The responses of plant photosynthesis to rapid fluctuations in environmental conditions are critical for efficient conversion of light energy. These responses are not well-seen laboratory conditions and are difficult to probe in field environments. We demonstrate an open science approach to this problem that combines multifaceted measurements of photosynthesis and environmental conditions, and an unsupervised statistical clustering approach. In a selected set of data on mint (*Mentha* sp.), we show that ‘light potentials’ for linear electron flow and non-photochemical quenching (NPQ) upon rapid light increases are strongly suppressed in leaves previously exposed to low ambient photosynthetically active radiation (PAR) or low leaf temperatures, factors that can act both independently and cooperatively. Further analyses allowed us to test specific mechanisms. With decreasing leaf temperature or PAR, limitations to photosynthesis during high light fluctuations shifted from rapidly induced NPQ to photosynthetic control of electron flow at the cytochrome *b_6_f* complex. At low temperatures, high light induced lumen acidification, but did not induce NPQ, leading to accumulation of reduced electron transfer intermediates, probably inducing photodamage, revealing a potential target for improving the efficiency and robustness of photosynthesis. We discuss the implications of the approach for open science efforts to understand and improve crop productivity.

## 1. Introduction

While oxygenic photosynthesis supplies energy to drive essentially all biology in our ecosystem, it involves highly energetic intermediates that can generate highly toxic reactive oxygen species (ROS) that can damage the organisms it powers [1]. Thus, the energy input into photosynthesis must be tightly regulated by photoprotective mechanisms that act at several key steps in the light reactions. The balance and kinetics of this regulation is an active target for crop improvement.

One class of photoprotective processes, known as non-photochemical quenching (NPQ), dissipates absorbed light energy as heat, thus diverting energy away from photosystem II (PSII) [2], decreasing the accumulation of reactive intermediates. This photoprotective capacity comes at the cost of decreased photochemical efficiency, and thus the organisms must regulate NPQ to balance the avoidance of photodamage with efficient energy conversion [3,4]. There are several forms of NPQ that differ in their mechanisms and rates of activation and deactivation. The most rapid NPQ form is qE, which is activated by acidification of the thylakoid lumen by the proton gradient (ΔpH) component of the thylakoid proton motive force (*pmf*) [2]. Lumen acidification activates the violaxanthin de-epoxidase or VDE [5–8] resulting in the conversion of violaxanthin (Vx) to antheraxanthin (Ax) and zeaxanthin (Zx); and protonation of PsbS, an antenna-associated protein required for qE [2], which appear to act cooperatively in setting the extent of qE. The conversion of Vx to Ax and to Zx is typically much slower than the rapidly reversible protonation of PsbS [2], and during prolonged illumination, the responses of qE will probably be limited by the rate of acidification and de-acidification of the thylakoid lumen, which are, in turn, governed by ion movements in the chloroplasts [9–11]. Slower forms of NPQ have also been demonstrated [12], including qI, which is related to the photodamage and repair of photosystem II (PSII) or qZ, which related to the accumulation of Zx (independently from qE) [13], qH, related to cold and high light stress [13], and qT, related to antenna state transitions [14].

The acidification of the thylakoid lumen also controls electron transfer at the cytochrome *b_6_f* complex, a process called photosynthetic control (PCON) [15–20], which prevents the build-up of electrons on the acceptor side of photosystem I (PSI) that can lead to photodamage [15,21–23]. Interestingly, PCON and qE (both responses to lumen acidification) are expected to have opposing effects on Q_A_ redox state. High levels of PCON in the absence of qE would lead to accumulation of plastoquinol (PQH_2_) and the reduced form of the PSII electron acceptor, Q_A_-, which can potentiate photodamage. Thus, these two processes must be tightly coordinated, with qE being activated at lumen pH somewhat less acidic than PCON [15].

Plants in natural environments are exposed to rapidly changing environmental conditions, especially light, which can change by orders of magnitude in less than a second. It has become clear that rapid and unpredictable fluctuations in light intensity can be more damaging than more gradual changes [22,24–32]. This sensitivity can partly be related to the build-up of reactive redox intermediates and thylakoid *pmf*, which can occur following low-to-high light transitions much more rapidly than the activation of photoprotective NPQ and PCON, leaving the photosynthetic apparatus prone to photodamage. Also, the slow recovery of NPQ following a decrease in light intensity can lead to substantial losses of photosynthetic efficiency [33].

Recently, it has been reported that engineering plants with increased expression levels of VDE and zeaxanthin epoxidase (ZE), resulted in accelerated formation and reversal of qE accompanied by increased plant productivity [3], suggesting that it may be possible to increase yield in crops by modifying photosynthetic regulatory responses.

On the other hand, we lack comprehensive surveys of the range of natural response of photosynthesis to real environmental fluctuations, in part because of a lack of deployable scientific equipment and methods to probe these processes in the field. Consequently, it has not been possible to assess the mechanistic bases of extant natural variations in these processes, their possible benefits or trade-offs, or which of these may be most useful for crop improvement.

Here, we introduce a method and proof-of-concept field data results to address the following questions: Can we assess the extent of natural variations in rapid responses to fluctuations in photosynthetically active radiation (PAR) intensity for both electron flow and photoprotection? How do these limitations depend on environmental conditions? What are the mechanisms that underlie these variations in responses to rapidly fluctuating light in the field?

We define the term ‘light potential’ (LP) as the plant to respond to sudden increases in PAR, either by using it for productive photochemistry or up-regulating photoprotective mechanisms. In effect, LP is the response of photosynthesis to removal of light limitations. Here, we introduce an approach to both measure and analyse these variations in LP, focusing on one species, *Mentha* sp*.*, under a limited set of conditions, and applied these to testing among a set of mechanisms for modulating that can be distinguished based on a range of optical measurements available using the MultispeQ 2.0 device, including: (i) PSI acceptor-side limitations to electron transfer; (ii) increased NPQ, which limits the input of light energy into photosystem II (PSII); and (iii) PCON, in which acidification of the lumen slows electron transfer at the level of plastoquinol (PQH_2_) oxidation by the cytochrome *b_6_f* complex.

The results show that the approach can effectively be used to assess the range of variations in LP under field conditions, as well as to test specific hypothetical models, setting up a broad-scale, multiple-participant, open science approach to exploring the responses across multiple species, genotypes and environments. The results also reveal, at least in *Mentha*, unexpected leaf temperature-dependent limitations in the rapid formation of NPQ that result in the accumulation of reduced PSII electron acceptor, Q_A,_ and a high thylakoid *pmf*, conditions likely to promote the formation of ROS.

## 2. Material and methods

### 2.1. Plants and leaf sampling

Measurements were made in a population of *Mentha spicata* (Spearmint) plants that have been maintained at the MSU Horticulture Gardens (East Lansing, MI, USA) for at least 10 years. The GPS locations of all measurements are included in the online dataset (https://photosynq.org/projects/rapid-ps-responses-pam-ecst-npqt-mint-dmk). Although it was not practical to exhaustively capture the lifecycle of the plants, the experimental strategy sampled a sufficiently wide range of conditions to allow clear patterns to emerge in the relationships between response behaviours and environmental parameters, as described below. The experiment took place over a 9-day experimental window between 21 July and 2 August 2019 (electronic supplementary material, figure S1A), sampling a range of times of day (between 5.30 and 18.40 local time), temperatures, etc. (electronic supplementary material, figure S1B). Measurements were made at multiple, alternating canopy levels and positions (subjectively at high, middle and low canopy levels) from early morning to later afternoon (electronic supplementary material, figure S1B), and at multiple locations across the plots on each day. Plants were up to 1 m in height. The leaf area index was not measured in this experiment. For the weather data, see the MSU Enviroweather website (https://mawn.geo.msu.edu/station.asp?id=msu, MSU Horticulture Teaching & Research Center, latitude: 42.6734, longitude: −84.4870, elevation: 264 m).

### 2.2. Measurements of photosynthetic and related parameters using MultispeQ 2.0

**Figure 1.**
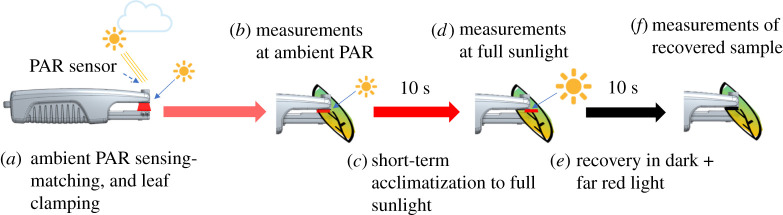
Experimental procedure of NPQ light potential designed to detect the change in NPQ induced under different light intensities. (*a*) A sensor on the MultispeQ continuously detects the ambient PAR in the field and reproduces this PAR value using an internal LED. (*b*) When the leaf clamp is closed over a leaf, the experiment begins by recording the local PAR, leaf temperature, ambient temperature, leaf angle and GPS position. After a short period of illumination at the measured ambient PAR, the first set of optical measurements are recorded. (*c*) Once completed, the leaf is exposed to a period of high PAR (2000 µmol m^−2^ s^−1^ equivalent to full sunlight) for 10 s. (*d*) The optical measurements are repeated at high PAR. (*e*) The leaf is then dark adapted (actinic light is switched off), with weak far-red background light for 10 s. (*f*) A final set of optical measurements is made to assess rapid dissipation of NPQ and reoxidation of accumulated reduced intermediates. Each set of optical measurements includes chlorophyll fluorescence and absorbance changes to give estimates of *Φ*_II_, LEF, NPQt, qL (Q_A_ redox state); ECSt, P_700_ redox state and g_H^+^_ (relative proton conductivity of the thylakoid ATP synthase), as described in Material and methods. Measurements taken at ambient and high light are designated with the subscripts amb and high, as in LEF_amb_, NPQ_amb_, qL_amb_ and LEF_high_, NPQ_high_, qL_high_.

Optical measurements were made using MultispeQ 2.0 hand-held instruments (https://photosynq.com), based on that presented by Kuhlgert *et al*. [34] and calibrated using the CaliQ calibration system (https://photosynq.com/caliq). The LP protocol used in the experiments can be found in the online project information (rapid-ps-responses-with-ecs-fast-ecs-dirk-and-npqt-dmk) as illustrated in figure 1. The protocol was designed to strike a balance among the needs for sampling large numbers of leaves, the desire for detailed spectroscopic measurements and the length of time the plant could be exposed to increased or decreased PAR. The full protocol, with measurements at ambient, after 10 s full sunlight and 10 s dark required about 35–40 s, at the limit of the time scale over which most researchers could steadily clamp a leaf in the instrument. The implications of the 10 s illumination and recovery time are discussed in the Results and Discussion sections.

In the first stage of the protocol (figure 1*a*), the MultispeQ was programmed to continuously (at about 5 Hz) measure PAR and reproduced these levels using a red actinic LED (655 nm emission peak) illuminating the adaxial surface of the leaf. When the MultispeQ detected that a leaf was clamped in the chamber, a series of measurement sequences was initiated. After a few seconds of illumination at ambient PAR (PAR_amb_) to allow for settling and setting of gains, the first set of measurements was made, estimating at PAR_amb_ linear electron flow (LEF) (LEF_amb_), NPQt (NPQ_amb_) and other photosynthetic parameters (figure 1*b*).

The actinic light was then increased to approximately full sunlight (2000 µmol m^−2^ s^−1^ red light) for 10 s (figure 1*c*), after which the photosynthetic measurements were repeated (figure 1*d*), yielding measurements of LEF_high_, NPQ_high_, etc. We chose full sunlight, rather than an artificially intense super-saturation light, to estimate LPs that could occur in the field, and not the absolute maximum, and to avoid non-physiological or photoinhibitory effects. Thus, the LPs of various processes will be limited as PAR_amb_ approaches full sunlight.

In the third stage of the experiment, the actinic light was then switched off, and a weak far-red light switched on for 10 s (figure 1*e*), following another repetition of the measurements to assess the extent of NPQ_t_ after relaxation (NPQ_rec_, figure 1*f*). Environmental parameters including PAR, temperature, humidity, leaf temperature, leaf angle and GPS location were measured either prior to or following the physiological measurements.

Chlorophyll fluorescence changes were measured using MultispeQ 2.0 devices to estimate PSII quantum efficiency (*Φ*_II_) and linear electron flow (LEF) [35,36], as well as qL, a measure of the fraction of Q_A_ in the oxidized state [37], and the extent of NPQ based on the rapid ‘total’ NPQ method developed by Tietz *et al*. [38], designated as NPQ_t_. Just prior to the saturation pulses, dark interval relaxation kinetics (DIRK, dark interval of approx. 300 ms) of the absorbance changes around 520 nm attributed to the electrochromic shift (ECS) were recorded. Fitting the ECS signals to exponential decay curves yielded estimates of the relative light–dark differences in thylakoid *pmf* (ECS_t_) and the proton conductivity of the chloroplast ATP synthase (*g*_H_+), as described in [16,39,40]. To account for differences in leaf thickness, light path or number of chloroplasts in various leaves, the ECS_t_ values were normalized to the relative chlorophyll contents as estimated by the SPAD parameter [34], which was measured at the end of the experiment. The extent of oxidation of P_700_ in the light was estimated by the DIRK of infrared LED light using an LED measuring pulse with peak emission at approximately 810 nm.

Two features of the instrument's leaf clamp provide free air flow to the outside, preventing the depletion of CO_2_ during the measurements. First, there is an approximately 3 mm space (or gap) between the leaf surface and the light guides, allowing lateral air flow. Second, this gap is connected to the external environment via a pair of rectangular (2 × 3 mm) air flow guides on the sides of the light guide, leading to the instrument case, the rear of which is open to the air. We thus do not expect any significant restriction of CO_2_ diffusion to the leaf surface. To check this, we compared measurements of 10 separate mint leaves made with an unmodified instrument with 10 made with an additional air pump that provided approximately 300 ml min^−1^ air exchange in the leaf clamp. We observed no significant differences (*p* > 0.5) in LEF values measured as above, under ambient (LEF_amb_) or high (LEF_high_, 2000 µmol m^−2^ s^−1^) red light, indicating that the air flow in the device was sufficient to prevent substantial CO_2_ restriction to the leaves.

### 2.3. Environmental conditions during light potential measurements in the field

Electronic supplementary material, figure S1A–C shows the distributions of environmental factors (light intensities, leaf temperatures) for the measurements analysed in this study. The MultispeQ sensor was positioned by the user to be parallel to the leaf surface, so that the cosine-corrected PAR sensor should effectively estimate PAR absorbed by the leaf surfaces *in situ* throughout their canopy, and thus the ambient PAR (PAR_amb_) values were dependent on both time of day (diurnal cycle, electronic supplementary material, figure S1B) and by leaf angle (electronic supplementary material, figure S1C). Ambient temperature and leaf temperatures (T_leaf_) were dependent on time of day, with obvious influences from weather-related fluctuations (electronic supplementary material, figure S1A, B). We chose to compare results with T_leaf_, rather than ambient temperature, to better reflect the effects on leaf photosynthetic processes. We note that previous results, e.g. Kuhlgert *et al*. [34], indicate that there may also be significant interactions between canopy position and photosynthetic parameters, though the current experiment did not explicitly record these positions, but rather sampled them as described in Material and methods.

### 2.4. Data calculations and cleaning

Data from the PhotosynQ platform were reprocessed and cleaned to improve the estimation of decay constants for ECS and near-infrared absorbance changes. As with any field experiments, some results were found to have obvious errors or be out of acceptable ranges, and were removed from the analysis. However, all original data were maintained in the online platform, allowing the reader to explore and reanalyse the effects of our data cleaning procedures. The rules and code for data flagging are defined in the Jupyter Notebook (see electronic supplementary material ‘Data Cleaning Notebook’). A total of 292 points were flagged from a total of 1346 original measurements. The majority of the flagged measurements (179) were due to a defective device. The remaining 113 flagged points can be attributed to user error (e.g. leaf movements during measurements) or poor signal-to-noise that resulted in parameter values outside the theoretical ranges.

## 3. Results

### 3.1. Field measurements of photosynthetic parameters under ambient and rapidly elevated PAR

**Figure 2.**
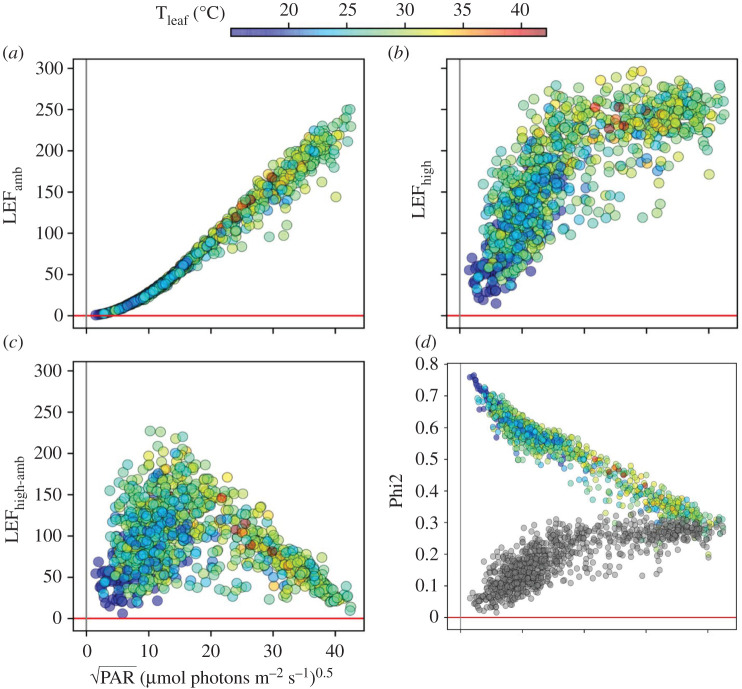
Light and temperature effects on LEF and photosystem II quantum efficiency (*Φ*_II_). Each parameter was plotted as a function of the square root of the ambient photosynthetically active radiation (PAR_amb_, X-axis) and leaf temperature (T_leaf_, coloration of points). (*a*) Dependencies of LEF measured at PAR_amb_; (*b*) LEF measured at 10 s high light (LEF_high_); (*c*) the high light-induced differences in LEF (LEF_high-amb_); (*d*) the PSII quantum efficiencies measured under ambient PAR (Phi2_amb_, points coloured by T_leaf_) and at 10 s high light (Phi2_high_, grey points).

Figure 2*a* shows LEF measured at PAR_amb_ (LEF_amb_) plotted against ambient PAR_amb_ and leaf temperature (T_leaf_, see coloration of points). The plots use the square root of PAR to better resolve the results at lower PAR_amb_, and to partially linearize the responses. LEF_amb_ increased with increasing PAR_amb_, with a roughly hyperbolic dependence and an apparent half-saturation point of about 350 µmol photons m^−2^ s^−1^, reaching maximum values of about 250 µmol electrons m^−2^ s^−1^ at 1700 µmol photons m^−2^ s^−1^.

Upon 10 s of exposure to 2000 µmol photons m^−2^ s^−1^ increased LEF to generally higher values (LEF_high_ figure 2*b*), indicating that LEF_amb_ was at least partly light-limited under all of the conditions. Note that each LEF_amb_ point was taken on different leaves at different times (Material and methods) and has corresponding LEF_high_ and LEF_high-amb_ measurements. The relationship between measurements is illustrated in electronic supplementary material, figure S2, which shows selected pairs of LEF_amb_ and LEF_high_ connected by vertical line segments. The extent of LEF_high_ was not uniform, but appeared to be strongly suppressed at low PAR_amb_ and/or low T_leaf_. The high light-induced difference in LEF (LEF_high-amb_) increased with PAR_amb_ at low light, reaching a peak at about 200 µmol photons m^−2^ s^−1^, above which it declined as PAR_amb_ approached PAR_high_ and LEF_high_ became light-saturated. The suppression of LEF_high_ was due to large decreases in the quantum efficiencies of PSII (Phi2, figure 2*d*). Phi2 at PAR_amb_ (Phi2_amb_) were highest at low PAR_amb_, and progressively saturated as light was increased. The opposite behaviour was seen with Phi2 measured after 10 s of high light (Phi2_high_ figure 2*d*, grey symbols) which was lowest at low PAR_amb_, and gradually increased with PAR_amb_.

### 3.2. Gaussian mixture model clustering analysis of field data

A simple linear effects model applied over the entire dataset (electronic supplementary material, table S1A) indicated strong correlations between LEF_amb_ and both PAR_amb_ and T_leaf_, suggesting that both environmental factors controlled LEF_amb_. However, such correlations may be coincidental since PAR and T_leaf_ are both expected to be dependent on weather or time of day, as is clear from the strong statistical correlations between PAR and T_leaf_. Also, the effects are likely to be co-dependent. For example, at low PAR_amb_, LEF_amb_ should be light-limited, and thus have minimal dependence on T_leaf_, but at higher PAR_amb_, may be more strongly controlled by temperature-dependent processes.

One approach to disentangling these effects would be to slice the data into segments, e.g. at different ranges of PAR_amb_, and test for correlations with T_leaf_ within each segment. However, arbitrarily chosen ranges for the segments can add bias, or fail to detect more complex interactions. We thus applied a Gaussian mixture model (GMM) clustering approach based on those presented earlier [41,42]. Because GMM is an unsupervised machine learning method, it can reduce bias in the selection of clusters that represent regions of distinct interactions among environmental and photosynthetic parameters. GMM assumes that the data points from the population of interest are being drawn from a combination (or mixture) of Gaussian distributions with certain parameters, and performs an optimization scheme to a sum of K Gaussian distributions, allowing for a completely unsupervised process, avoiding potential user bias. An expectation–maximization (EM) algorithm was used to fit the GMM to the dataset, generating a series of Gaussian components (clusters) with distributions characterized by specific means and covariance matrices. The optimal number of clusters was determined using the Bayesian information criterion (BIC), the value of the maximized log likelihood, with a penalty on the number of parameters in the model [41–44]. This approach also allows comparison of models with differing parametrizations and/or differing numbers of clusters, because the volumes, shapes and orientations of the covariances can be constrained to those described by defined models [41].

Clusters obtained through GMM have both within cluster (intracluster) and between cluster (intercluster) variations. In order to test for intercluster variation, we used the clustering assignment obtained for one parameter (or response) and applied it on another response. Here we want to investigate what would be the distinctive behaviour of different responses if we have used the same configuration. Using the same set of cluster assignments to different responses, one might be skeptical of the clustering behaviour as responses interact differently with PAR_amb_ and T_leaf_. In that case, we might not be able to directly compare the intercluster behaviours of responses. To mitigate this issue, we use the GMM clustering as a tool to create a ‘baseline’ clustering configuration for one response and use that configuration over other responses. We set up our hypothesis as two responses are similar under the same configuration against they are not. If the interaction pattern of one response with PAR_amb_ and T_leaf_ changes over the other response, we reject our hypothesis and imply that different configurations of PAR_amb_ and T_leaf_ interact differently with responses. By doing this we are able to disentangle the effect of PAR_amb_ and T_leaf_ and infer regarding the intracluster variations as to be a key element to determine variations in the interactions between parameters and variations in environmental conditions, e.g. to assess if a relationship is modulated in different ways under different ranges of conditions. Also, as will be seen in the Discussion, intercluster variations (differences in the mean and covariances between clusters) can be used to differentiate distinct patterns of behaviour, or mechanistic interactions, between conditions.

As shown in electronic supplementary material, figure S3, GMM analysis of LEF_amb_, PAR_amb_ and T_leaf,_ found six distinct, compact clusters that differed in the mode of interaction among the photosynthetic and environmental parameters. Encompassing points with lower PAR_amb_ showed strong (clusters 1, 2, 4 and 5) dependence of LEF_amb_ on PAR_amb_, with little contributions from T_leaf_. By contrast, two clusters (3 and 6), which included points at higher PAR_amb_, showed substantial dependencies on both PAR_amb_ and T_leaf_. These results are consistent with LEF being predominantly light-limited at low ambient PAR, but increasingly limited by temperature-dependent processes at higher PAR. The presence of these two classes of clusters indicates that PAR_amb_ and T_leaf_ are likely to affect LEF_amb_ in independent ways. The fact that the shapes of the clusters were not determined with individual slicing under the individual parameters for PAR_amb_ and T_leaf_, but with a co-dependence on both PAR_amb_ and T_leaf_, suggests that, under some conditions, these effects interact, e.g. T_leaf_ may affect the dependence of LEF_amb_ on PAR_amb_.

GMM identified five distinct clusters for interactions among LEF_high_, PAR_amb_ and T_leaf_ (electronic supplementary material, figure S4). In contrast to the results on LEF_amb_, clusters at lower PAR_amb_ (1, 2 and 4) showed LEF_high_ dependencies on both T_leaf_ and PAR_amb_, while cluster 3 showed correlations with T_leaf,_ but not with PAR_amb_. The stronger dependence on T_leaf_ of LEF_high_ compared with LEF_amb_ implies that the exposure to high light revealed additional rate limitations in LEF_high_ that were more strongly controlled by both T_leaf_ and PAR_amb_ and that, at least under some conditions, these effects were independent of each other.

### 3.3. Analysis of NPQ

**Figure 3.**
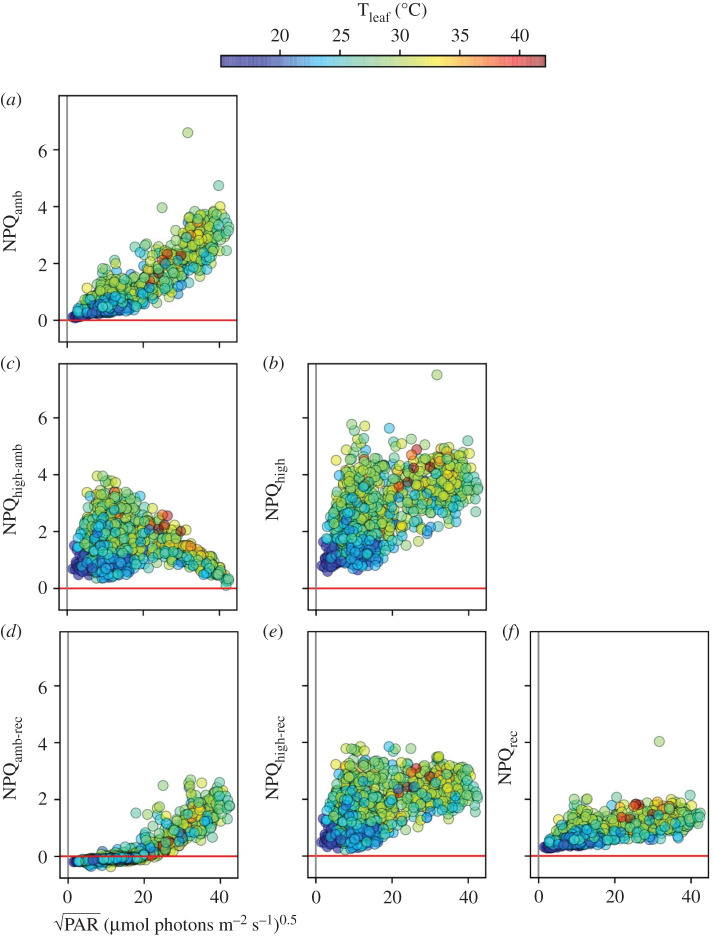
Light and temperature effects on NPQ. The NPQ parameter was plotted as functions of the square root of the ambient photosynthetically active radiation (PAR_amb_, X-axis) and leaf temperature (T_leaf_, coloration of points). (*a*) Induced NPQ measured at PAR_amb_; (*b*) NPQ measured at 10 s high light (NPQ_high_); (*c*) the high light-induced differences in NPQ (NPQ_high-amb_); (*d*) the difference in induced NPQ level at ambient PAR and the 10 s recovery time in the dark (NPQ_amb-rec_); (*e*) the difference in induced NPQ level at 10 s high PAR and the 10 s recovery time in the dark (NPQ_high-rec_); (*f*) the NPQ level after 10 s in the dark (NPQ_rec_).

NPQ_t_ measured under PAR_amb_ (NPQ_amb_, figure 3*a*) showed a positive correlation to PAR_amb_, with an apparent tendency for smaller values at lower T_leaf_. NPQ_amb_ showed considerable variations, compared with LEF_amb_, even at low PAR_amb_, consistent with the idea that NPQ is governed not only by PAR but by metabolic, developmental or other environmental parameters.

Figure 3*b* shows NPQ_t_ values measured at 10 s full sunlight (NPQ_high_). The NPQ LP, or light-induced differences in NPQ (NPQ_high-amb_) are shown in figure 3*c*. While NPQ_high-amb_ was always positive, both NPQ_high-amb_ and NPQ_high_ were suppressed at low PAR_amb_ or T_leaf_. NPQ_t_ measured after the 10 s dark recovery period (NPQ_rec_, figure 3*f*) was consistently lower than NPQ_amb_ and NPQ_high_. The difference between NPQ_amb_ and NPQ_rec_ (NPQ_amb-rec_, figure 3*d*) ranged from slightly negative at low PAR_amb_, where the majority of NPQ_amb_ was rapidly reversible, to about one at the higher PAR_amb_, where about half of NPQ_amb_ was rapidly reversed.

Overall, these results indicate that a large fraction (in many cases the majority) of NPQ_amb_ as well as NPQ_high_ recovered within 10 s of darkness and can probably be attributed to qE, and thus, under our conditions, qE is likely to be the most important form of NPQ for rapid adjustments to photoprotection. The residual, more slowly reversible, components reaching a little above 2 are likely to include qI or qZ [45,46], although the limited time frame for the protocol does not allow us to rule out contributions from longer-lived qE. It is also important to consider that the fraction of light energy dissipated by the NPQ, i.e. *Φ*_NPQ_, will also depend on the fraction of PSII in open states [37], which will also be impacted by ambient and fluctuating light, T_leaf_ and other factors.

As with LEF, a simple linear effects model (electronic supplementary material, table S1B) showed strong interactions between T_leaf_ and PAR_amb_, on NPQ_amb_, and the corresponding GMM analysis identified four clusters (electronic supplementary material, figure S5). Cluster 1, which encompassed the lowest range of PAR_amb_ values, showed strong dependence on PAR_amb_, with no significant dependence on T_leaf_. The remaining clusters showed either dependence solely on T_leaf_ (cluster 4) or co-dependence on PAR_amb_ and T_leaf_ (clusters 2 and 3). Because GMM clustering suggests that T_leaf_ and PAR_amb_ can interact or act independently, depending on conditions, we excluded the linear effects models and focused on GMM for analyses of the remaining parameters.

For the analysis of NPQ_high_ (electronic supplementary material, figure S6), we used the clusters found for NPQ_amb_ (electronic supplementary material, figure S5), allowing us to directly compare changes in correlations among parameters within each cluster [41]. Cluster 1, which encompassed the lowest range of PAR_amb_ values, showed strong dependence of NPQ_high_ on both PAR_amb_ and T_leaf_. This pattern of dependencies was in contrast to that for cluster 1 for NPQ_amb_, which showed dependence solely on PAR_amb_. At a higher range of PAR_amb_ (cluster 3), NPQ_high_ showed significant dependence solely on T_leaf_, again in contrast to the corresponding cluster for NPQ_amb_, which showed dependencies on both PAR_amb_ and T_leaf_. Overall, compared with NPQ_amb_, NPQ_high_ showed increased dependence on T_leaf_ in all clusters, suggesting that it is more substantially controlled by metabolic or physiological factors (see Discussion).

### 3.4. The redox state of Q_A_

**Figure 4.**
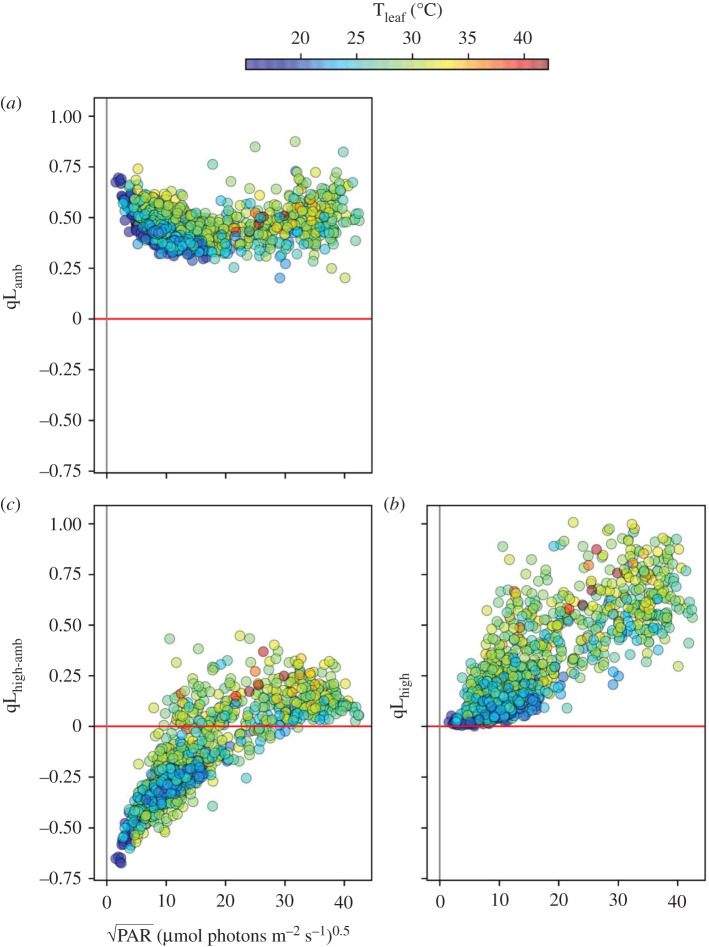
The light and temperature dependencies of the redox state of Q_A_. The qL parameter, a measure of fraction of Q_A_ in its oxidized state, was measured as described in Material and methods, (*a*) under ambient light (qL_amb_), (*b*) at 10 s of high light (qL_high_), and (*c*) the change in qL between high and ambient PAR (qL_high-amb_) as a function of the square root of ambient PAR.

Figure 4*a* shows the dependencies of Q_A_ redox state (qL) on PAR and T_leaf_. qL measured at PAR_amb_ (qL_amb_, figure 4*a*), was relatively constant (ranging from about 0.3 to 0.75) across PAR_amb_, with somewhat higher values at both extremes of PAR_amb_. Lower leaf temperatures appeared to be associated with lower qL values, over the entire range of PAR_amb_, although the effect was particularly pronounced at low light. By contrast, qL measured at 10 s of high light (qL_high_, figure 4*b*) showed strong dependence on PAR_amb_, ranging from near zero (fully reduced Q_A_) at low PAR_amb_, to almost one (fully oxidized) at higher PAR_amb_. Again, low T_leaf_ appeared to correlate with lower qL_high_ throughout the range of PAR_amb_. Strikingly, as shown in figure 4*c*, the high light treatment induced two distinct effects: at low PAR_amb_ and/or T_leaf_, it induced a net reduction of Q_A_, while it had the opposite effect at higher PAR_amb_ and T_leaf_.

GMM clustering for qL_amb_, PAR_amb_ and T_leaf_ (electronic supplementary material, figure S7) identified four distinct clusters. In cluster 2, which encompasses points at low PAR_amb,_ significant associations were observed only between qL_amb_ and PAR_amb_. Clusters 1, 3 and 4 (at higher PAR_amb_) showed co-dependencies between qL_amb_ and both PAR_amb_ and T_leaf_. GMM clustering for qL_high_, PAR_amb_ and T_leaf_ showed five distinct clusters (electronic supplementary material, figure S8). Clusters 1, 2 and 5, which encompassed generally lower ranges for PAR_amb_ and T_leaf_, showed qL_high_ dependencies on both PAR_amb_ and T_leaf_. Clusters 3 and 4 (generally with higher PAR_amb_ and T_leaf_ values) showed only dependencies on T_leaf_. The overall pattern of cluster behaviour was similar to that observed with respect to NPQ_amb_ and NPQ_high_.

### 3.5. P700 redox state

**Figure 5.**
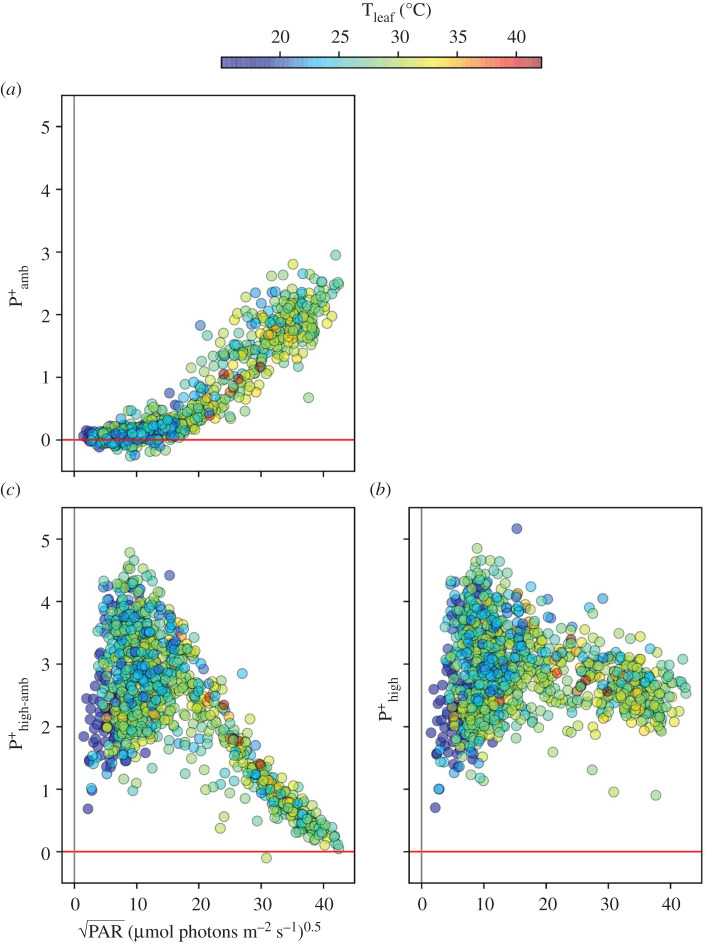
The light and temperature dependencies of the redox state of P_700_^+^. The redox state of P700 was measured using DIRK at 810 nm absorbance change (*a*) under ambient light (P^+^_amb_), (*b*) at 10 s of high light (P^+^_high_), and (*c*) the change in P^+^ between high and ambient PAR (P^+^_high-amb_) as a functions of the square root of ambient PAR.

Figure 5 shows the extent of oxidized P_700_^+^ (P^+^), based on the DIRK of absorbance changes at 810 nm. P_700_^+^ at PAR_amb_ (P^+^_amb_, figure 5*a*), after 10 s of high light (P^+^_high_, figure 5*b*) and the light-induced difference (P^+^_high-amb_, figure 5*c*). The extent of P^+^_amb_ was nearly linearly related to PAR_amb_. Increasing the light resulted in higher P^+^ values (P^+^_high_), indicating that, in all cases, PSI became more oxidized at high light. The extent of the light-induced oxidation was dependent on PAR_amb_, with lower extents at low PAR_amb_, and a peak at about 200–300 µmol photons m^−2^ s^−1^. The decrease at higher PAR_amb_ was probably due to the accumulation of pre-oxidized P_700_ prior to the high light treatment.

The full extent of P^+^_high_ was relatively constant over the conditions, suggesting that high light was able to nearly fully oxidize P_700_. However, there was a slight trend to lower P^+^_high_ at the highest PAR_amb_ or T_leaf_, suggesting that total oxidizable PSI may have decreased at high light or temperatures, perhaps reflecting accumulation of PSI photodamage or electron sink limitations. Consistent with these general trends, GMM analyses of P^+^_amb,_ PAR_amb_ and T_leaf_ identified four distinct clusters (electronic supplementary material, figure S9), with dependencies on either PAR_amb_ by itself (clusters 3 and 4), or both PAR_amb_ and T_leaf_ (clusters 1 and 2). GMM clustering for P^+^_high_ identified five distinct clusters (electronic supplementary material, figure S10), that showed a positive dependency of P^+^_high_ on either PAR_amb_ (cluster 1), or T_leaf_ (cluster 5), or a small, negative dependence on T_leaf_ (cluster 3).

### 3.6. ECSt and thylakoid *pmf*

**Figure 6.**
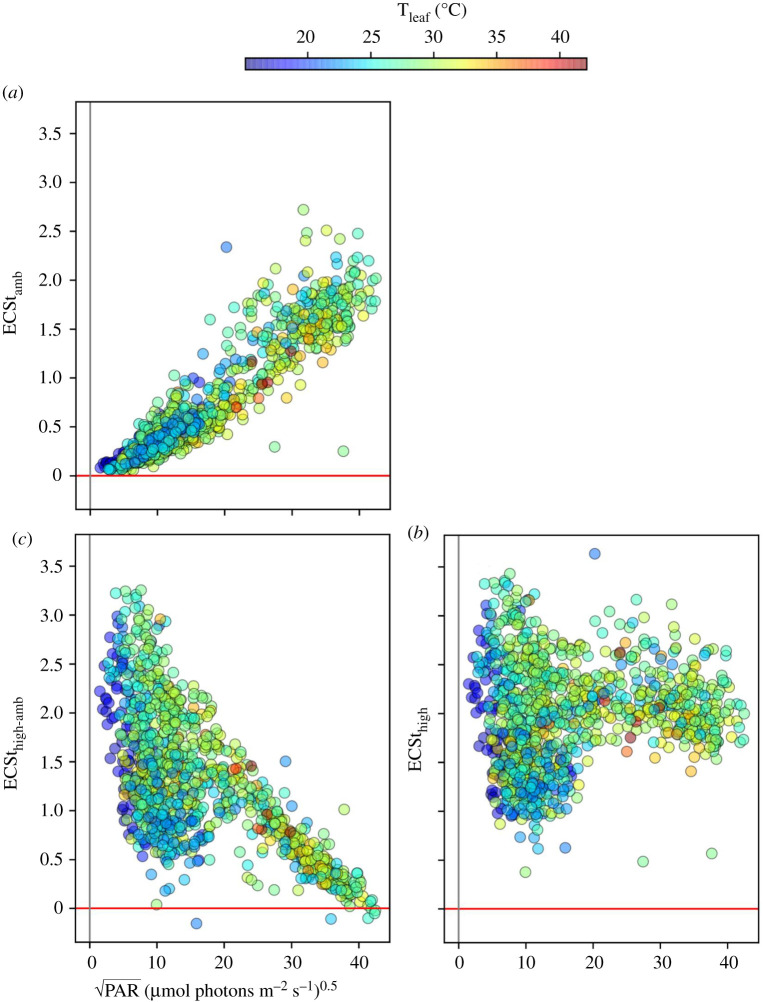
The light and temperature dependencies of the thylakoid *pmf* probed using ECSt signal. The *pmf* was measured using ECS (*a*) under ambient light (ECSt_amb_), (*b*) at 10 s of high light (ECSt_high_), and (*c*) the change in ECSt between high and ambient PAR (ECSt_high-amb_) as a functions of the square root of ambient PAR.

Figure 6 shows dependencies of relative thylakoid *pmf*, estimated by normalized ECSt measurements, at ambient PAR (ECSt_amb_, figure 6*a*) and after 10 s exposure to high light (ECSt_high_, figure 6*b*). The high light-induced differences (ECSt_high-amb_) are shown in figure 6*c*. ECSt_amb_ showed strong, positive correlations with PAR_amb_, similar to the responses of NPQ_amb_ (figure 3*a*) and P^+^_amb_ (figure 5*a*). ECSt_high_ values were, in general, larger than ECSt_amb_, resulting in positive values for ECSt_high-amb_. At low PAR_amb_, ECSt_high_ showed high variability, suggesting that the response is strongly dependent on other factors, but appeared to saturate (flatten) at higher PAR_amb_. These behaviours were reflected in ECSt_high-amb_, which showed strong variability at lower PAR_amb_ or T_leaf_, peaked at about 50–100 µmol photons m^−2^ s^−1^, and saturated at higher PAR_amb_.

GMM analysis of ECSt_amb_ identified five distinct clusters (electronic supplementary material, figure S11). The cluster at the lowest range of PAR_amb_ (cluster 1) showed dependence primarily on PAR_amb_. The remaining clusters showed positive correlations between ECSt_amb_ and PAR_amb_, but negative correlations with T_leaf_. By contrast, GMM of ECSt_high_ (electronic supplementary material, figure S12) showed almost no dependence on either PAR_amb_ or T_leaf_, except at the lowest PAR_amb_ (cluster 1) which showed negative correlations with PAR_amb_ and positive correlations with T_leaf_.

## 4. Discussion

### 4.1. Using PhotosynQ and MultispeQ to sample and resolve the effects of environmental fluctuations on photosynthetic processes

The MultispeQ measurements described above were designed to explore the photosynthetic responses of plants in a natural, fluctuating environment. In this type of field experiment, it is not possible to control all variables. Rather, the strategy was to ‘sample’ responses under as many conditions as practical, while recording key metadata so that subsequent analyses can assess the impacts of various environmental fluctuations. Thus the observed trends may reflect both primary and acclimatory factors that change (or accumulate) over different time scales. Correlations that appear in such analyses can be used to test, at least to some extent, certain models, though it is important to note that more controlled experiments will be needed to fully determine cause–effect relationships, as discussed below.

A major outcome of the experiment is that, despite the fact that measurements were made over many plants, times, etc., clear patterns of responses emerged that allow us to make some broad conclusions about the responses of photosynthesis to ambient and rapidly changing light. For example, the majority of NPQ_high_ was found, in general, to be rapidly reversible, suggesting that qE was the major contributor: at lower PAR_amb_, that majority of NPQ_high_ was rapidly induced (figure 3*c*), while at higher PAR_amb_, pre-existing NPQ was rapidly recoverable (figure 3*e*).

Another important trend was the suppression of the LPs of both LEF (figure 2) and NPQ (figure 3) under some conditions, particularly under lower PAR_amb_ and/or T_leaf_. Further, strong decreases in LEF_high_ were not always accompanied by compensatory increases in NPQ_high_, implying that the productive and photoprotective LPs can be simultaneously suppressed under certain conditions, a situation that is likely to promote the formation of ROS and photodamage (see also below), with important implications for understanding the environmental robustness of photosynthesis [29].

### 4.2. Disentangling interacting environmental impacts on photosynthetic processes

A key challenge to the field experiment approach is in teasing apart effects from different environmental factors, especially considering that such factors may be co-dependent or interact with each other in complex ways. For example, in visual inspection, most of the parameters show apparent dependencies on both PAR_amb_ and T_leaf_ (e.g. figures 2–6) but, because increases in T_leaf_ are often correlated with increases in PAR_amb_, the effects of the two parameters may have been coincidental. It may also be that the environmental parameters interacted in complex ways, e.g. high PAR_amb_ may have exacerbated the effects of low T_leaf_. To address these issues, we applied an approach based on GMM to identify clusters representing distinct interactions among parameters. The approach is unsupervised, thus eliminating potential bias, while allowing us to test for changes in the environmental dependencies among multiple environmental parameters (electronic supplementary material, figures S3–S12).

Analysis of GMM clusters implied that most parameters were dependent on both PAR_amb_ and T_leaf_, and at least under some conditions these effects are independent, or that one of the two factors predominates. Thus, the effects cannot be explained simply by coincidences between increased PAR and temperatures. Moreover, the non-rectilinear shapes of the clusters suggest that the effects of PAR_amb_ and T_leaf_ were interactive, e.g. changes in T_leaf_ modulated the effects of PAR_amb_ and vice versa. Overall, these interactions are in line with well-known temperature and PAR dependence of photosynthesis, but this type of analyses can reveal the specific combination of conditions that induce distinct behaviours, allowing for assessments of the involvement of specific mechanisms (see below) and to identify genotypic or management impacts on crop resilience and productivity.

At low PAR_amb_, we expect steady-state photosynthesis to be predominantly light-limited, and thus the effects of T_leaf_ should be low. As light increases, downstream biochemistry should become increasingly limiting. Because downstream energy storage and metabolic processes are likely to be more temperature dependent than photochemistry, this shift may allow us to distinguish between these types of limitations. Such behaviours are apparent in many of the measured parameters, e.g. LEF_amb_, which was not substantially dependent on T_leaf_ at low PAR_amb_, but became co-dependent on PAR_amb_ and T_leaf_ at higher PAR_amb_ (figure 2*a*; electronic supplementary material, figure S3), consistent with a progressive shift from light-limitation to assimilation-limitation. Similarly, NPQ_amb_ was solely dependent on PAR_amb_ in the cluster at low PAR_amb_, but became increasingly dependent on T_leaf_ as PAR_amb_ increased (figure 3*a*). This shift is consistent with a control of NPQ_amb_ by PAR (at low PAR_amb_) and downstream metabolic processes, particularly at higher PAR_amb_, e.g. due to regulation of the ATP synthase activity or cyclic electron flow (CEF) [47].

By contrast, LEF_high_ and NPQ_high_ showed much greater dependence on T_leaf_, and these differences were more pronounced when the high light was imposed on leaves at low PAR_amb_ and T_leaf_, i.e. the opposite of what was seen for LEF_amb_ and NPQ_amb_. Interestingly, the LEF_high_ rates achieved in leaves exposed to lower PAR_amb_ were strongly suppressed below the maximum LEF_amb_ values measured at higher PAR_amb_ (compare figure 2*a,b*), This behaviour suggests that the suppression of LEF_high_ occurs when abrupt increases in light overwhelm the activation of downstream energy storage and metabolic processes. This is generally consistent with observations that the activities of metabolic enzymes are regulated to match the availability of energy from the light reactions, which involve a large suite of co-regulatory processes, as extensively reviewed elsewhere, (e.g. [16,47–53]), but that these responses lag behind the changes in light. The *in situ* LP measurements afforded by MultispeQ show that these situations are very likely to occur under many field situations.

These results also imply that accurate estimates of LEF, NPQ and other photosynthetic parameters *under natural conditions* will require measurements under ambient light, because sudden changes in PAR can lead to severe perturbations in photosynthetic limitations or regulation. Attempts to ‘simplify’ field experiments by setting PAR to some constant value will lead to strong perturbations and the measured values will reflect these perturbations. The effects are vividly demonstrated by the opposite dependencies of Phi2_amb_ and Phi2_high_ on PAR_amb_ (figure 2*d*), and validate the use of the PAR matching feature of the MultispeQ instrument. Nevertheless, as shown here, the effects of these perturbations can be informative, but care must be taken in extrapolating to the non-perturbed state. It is also important to keep in mind that the rates of acclimatization may vary substantially between species, and that these may be assessed by performing more intensive experiments with variable high light and dark recovery times.

### 4.3. Mechanisms for controlling the light potentials of LEF and NPQ using MultispeQ field data

The rapid reversal of NPQ_amb_ and NPQ_high_ over 10 s of dark indicated that, under our conditions, a large fraction of NPQ is in the form of qE (figure 3*b,c*), and thus dependent on lumen acidification and subsequent pH-dependent responses. It is important to note, though, that residual NPQ will contribute to decreases in photochemical efficiency, and that the extent of these effects will also be impacted by other factors, including the redox state of Q_A_ [37]. Lumen acidification can be controlled by changes in proton influx (through changes in LEF and CEF), proton efflux through the ATP synthase and the partitioning of *pmf* into electric field (Δ*ψ*) and ΔpH components, which in turn, are impacted by metabolic status, as proposed earlier [15,39]. Here, we explore the possible mechanistic bases for these effects, by comparing the correlations among MultispeQ measurements.

**Scheme 1.**
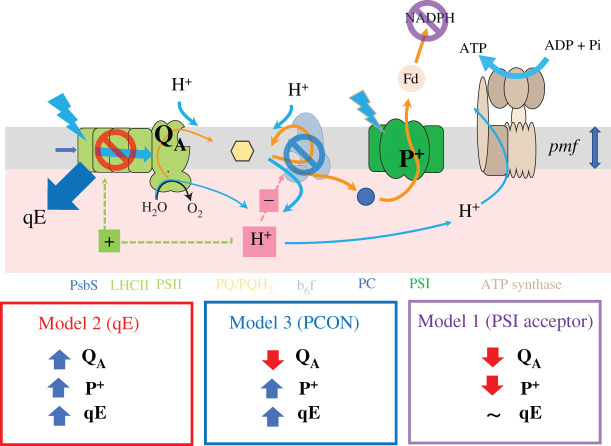
Three basic mechanistic models describing proposed processes that can limit the LPs of photosynthetic and photoprotective mechanisms.

Scheme 1 illustrates three basic mechanistic models describing proposed processes that can limit the LPs of photosynthetic and photoprotective mechanisms. The models make qualitative predictions about how the actions of each mechanistic model will impact correlations between measured photosynthetic parameters, and thus can be used as a framework for interpreting the field data introduced in Results. The expected effects on the measured parameters are summarized in scheme 1, which shows specific effects of each model.

**Model 1: PSI acceptor-side limitations** (scheme 1, Model 1) where lack of NADP^+^, ferredoxin or other PSI acceptors prevent further LEF. We expect this limitation to result in accumulation of electrons throughout the electron transfer chain, thus resulting in net reduction of Q_A_ (decreasing qL) and P_700_^+^ (decreasing the 810 nm absorbance signal). The decreases in proton fluxes associated with back-up of electrons may, in addition, prohibit rapid, light-induced increases in *pmf*, lumen acidification and qE responses.

**Model 2: Increased NPQ** (scheme 1, Model 2) should decrease delivery of excitation energy to PSII (but not to PSI), resulting in net oxidation of Q_A_ (increasing qL) and P_700_^+^ (increased 810 nm DIRK signal). Under some conditions, the NPQ will be rapidly induced by increased *pmf* and lumen acidification followed by activation of qE, which should be visible as increased NPQ_high-amb_. Under other conditions, e.g. at higher PAR_amb_, NPQ may already have been induced. If this NPQ is in the form of rapidly reversible qE, it should substantially decay during the 10 s dark recovery period, resulting in increased NPQ_high-rec_. More slowly induced or relaxing forms of NPQ, including qI, qZ and long-lived qE, may be also present prior to and throughout the experiment. The forms should register as increases in NPQ_rec_, but not in NPQ_high-amb_ or NPQ_high-rec_, but given that the high light and recovery periods were only 10 s long, our results do not allow us to distinguish among these possible forms.

**Model 3: Photosynthetic control** (PCON, scheme 1, Model 3). PCON results from the slowing of PQH_2_ oxidation at the cytochrome *b_6_f* complex as the lumen becomes acidified. If PCON occurs without activation of qE, we expect a net reduction of Q_A_ (decreasing qL) but a net oxidation of P^+^ (increasing the 810 nm absorbance signal).

The qE and PCON models can be further subdivided [15,18]. In most cases, we expect lumen acidification accompanied by elevated *pmf*, reflected in an increased ECSt signal, which can be induced by increased proton influx into the lumen, due to increased LEF, increased CEF, or decreased conductivity of the thylakoid to protons (*g*_H_^+^) by slowing the ATP synthase, all of which can contribute to change in *pmf* under fluctuating light [11,18,54,55]. Alternatively, lumen acidification can also be associated with an increase in the fraction of *pmf* that is stored as ΔpH, by controlling the flow of counterions across the thylakoid membrane, altering the partitioning of *pmf* in ΔpH and Δ*ψ* [10,16,56]. In this case, acidification may occur with little or no increases in total *pmf*, or the rates of proton influx [57], though the current field-based data do not allow us to directly distinguish these possibilities.

These models, while not mutually exclusive, will tend to counteract each other, at least within a particular leaf. For instance, PSI acceptor-side limitations will tend to inhibit electron flow, thus decreasing proton flux and *pmf* generation. On the other hand, the generation of *pmf* will tend to slow electron flow (through Models 2 or 3), thus preventing the build-up of electrons on PSI electron acceptors. However, it is important to note that, in a survey-type experiment like ours, photosynthesis in different leaves may be limited by distinct processes, and thus any collection of samples may reflect various combinations of the above models.

### 4.4. Testing models for limitations in light potentials

**Figure 7.**
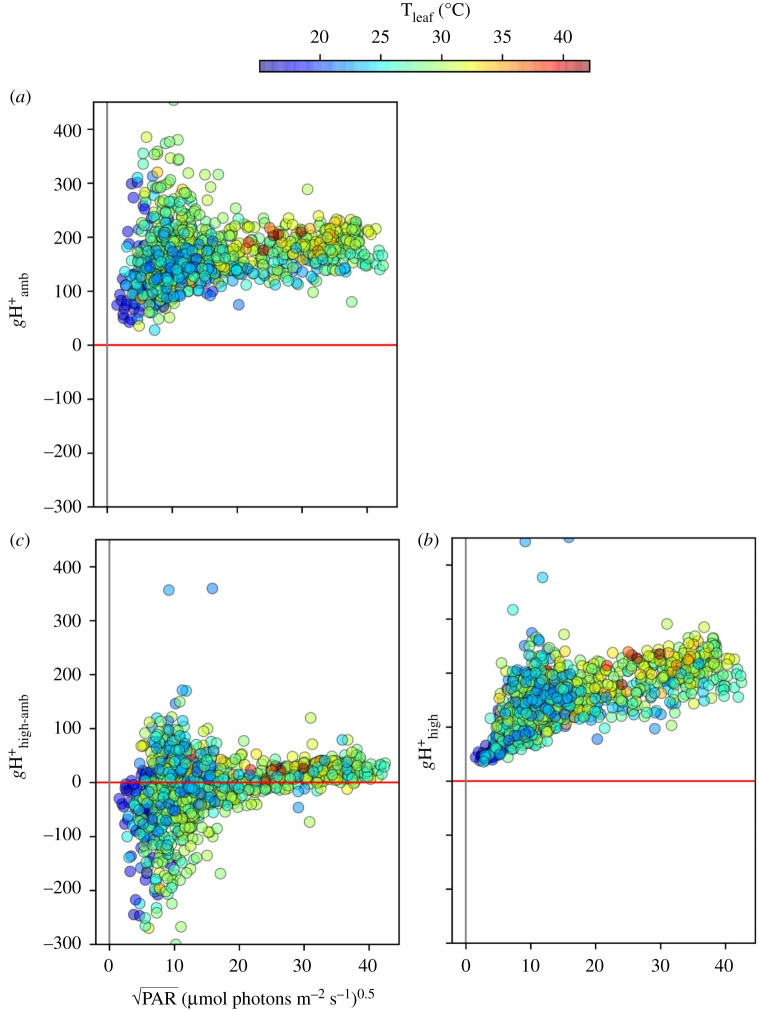
The relationship between light-induced thylakoid *pmf* and changes in P_700_ redox state. Changes in the thylakoid *pmf* (ECSt_high-amb)_ were estimated using the ECSt parameter, and changes in P_700_^+^ were measured using the absorbance changes at 810 nm, as described in Material and methods, under ambient light (ECSt_amb_, P^+^_amb_) and after 10 s of high light (ECSt_high,_ P^+^_amb_). The coloration of the points was set to a function of the square root of ambient PAR (PAR_amb_).

By plotting MultispeQ parameters against each other, we can test for more detailed patterns of behaviours predicted by the above models. Figure 7 shows that P^+^_high-amb_ (high light-induced P_700_ oxidation) was positively correlated with light-induced increases in *pmf* (ECSt_high-amb_). Under all conditions, increasing PAR from PAR_amb_ to PAR_high_ resulted in a net oxidation of P_700_, i.e. P^+^_high-amb_ was consistently positive. This behaviour is consistent with Models 2 (NPQ) or 3 (PCON), both of which predict a decrease in delivery of electrons from PSII to PSI. By contrast, we did not see evidence for high light-induced net *reduction* of P_700_^+^, i.e. values of negative P^+^_high-amb_, implying that Model 1 was not a major limitation to LEF LP. This does not exclude Model 1 from limiting photosynthesis in different species and conditions, as has been proposed to be important in chilling sensitive plants [58] as well as under pulse light [59] or in mutants that sufficiently acidify the lumen and activate PCON [21,23]. The apparent avoidance of Model 1 (or prevalence of Models 2 and 3) behaviour may reflect the ‘tuning’ of the light reactions to prevent the accumulation of reduced electron acceptors of PSI associated with photodamage [23], and the associated O_2_ caused by build-up of electrons on PSI [60].

Overall, the behaviours seen in figure 7 are consistent with restrictions in electron flow to PSI imposed by increases in *pmf*, most likely through the acidification of the thylakoid lumen. In the case of Model 2 (rapid NPQ), this would be related to the induction of qE, while in Model 3 (PCON), this could be related to slowing of electron flow at the cytochrome *b_6_f* complex.

**Figure 8.**
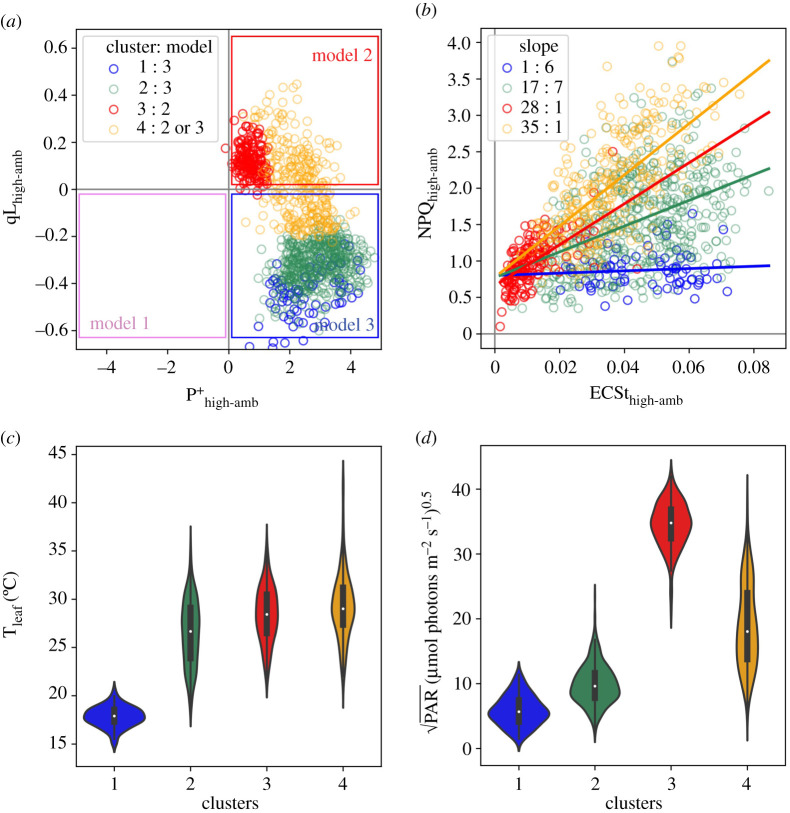
Relationships among measured parameters, predicted model behaviours and clustering. The relationships between light-induced changes in Q_A_ redox state and P700 redox state (*a*), and between rapidly inducible NPQ and thylakoid *pmf* (*b*) and the leaf temperature (*c*) and PAR (*d*) dependencies of Gaussian mixture models (GMM) clusters. Changes in P700^+^ (P^+^_high-amb_)_,_ Q_A_ redox state (qL_high-amb_), rapid changes in NPQ (NPQ_high-amb_) and thylakoid *pmf* (ECSt_high-amb_) were measured as described in Material and Methods. Data were clustered using the GMM approach described in the text, resulting in four distinct clusters, designated by the blue, green, red and ochre symbol colours (see legend in *a*). In (*b*), the slopes for the relationship between NPQ_high-amb_ and ECSt_high-amb_ were estimated by linear regression (slopes for clusters 1, 2, 3 and 4 were estimated to be 1.6, 17.7, 28.1 and 35.1, respectively). Panels (*c,d*) show distributions of (*c*) leaf temperatures (T_leaf_) and (*d*) square root of ambient PAR for each cluster in (*a,b*).

Figure 8*a* further investigates this behaviour by plotting the dependence of high light-induced changes in P_700_^+^ (P^+^_high-amb_) with changes in Q_A_ redox state (qL_high-amb_). The expected theoretical changes in measurable parameters upon activation of the three models are indicated by the coloured boxes in the figure, and can be related to Models 1–3 in scheme 1:

— **Model 1** (violet box) predicts net **reduction** of P_700_ (P^+^_high-amb_ < 0) and net **reduction** of Q_A_ (qL_high-amb_ < 0)
— **Model 2** (red box) predicts net **oxidation** of P_700_ (P^+^_high-amb_ > 0) and net **oxidation** of Q_A_ (qL_high-amb_ > 0)
— **Model 3** (blue box) predicts net **oxidation** of P_700_ (P^+^_high-amb_ > 0) but net **reduction** of Q_A_ (qL_high-amb_ < 0).

We observe behaviours consistent with both Models 2 and 3, suggesting that the behaviour of the system changed with conditions. Note that the boxes in figure 8*a* represent ‘pure’ behaviours, and it is possible that the effects of a particular mechanism may be intermediate, e.g. the responses may be limited by a combination of reduction of Q_A_ and increased NPQ.

Figure 8*b* plots the dependence of NPQ_high-amb_, which can be attributed to light-induced qE changes, on light-induced *pmf* changes (ECSt_high-amb_). A generally positive correlation was observed between NPQ_high-amb_ and ECSt_high-amb_, but with high variability, especially at higher values. Applying the clustering obtained for figure 8*a* on top of the data in figure 8*b*, we see that this variability can be explained by the environmental conditions and the modes of behaviours. Specifically, we see clear evidence for condition-dependent suppression of rapid activation of qE in response to increases in *pmf*. Particularly, the sensitivities of NPQ_high-amb_ to ECSt_high-amb_, as indicated by the slopes in figure 8*b*, were smallest in clusters 1 (slope ∼ 1.6) and 2 (slope ∼ 17.7), which comprise those with Model 3-like behaviour and occurred at low T_leaf_ and PAR_amb_ values. Higher sensitivities of NPQ_high-amb_ to ECSt_high-amb_ were seen for clusters 3 (slope ∼ 28.1) and 4 (slope ∼ 35.1), which comprised those associated with Models 2 and intermediate, and occurred at higher T_leaf_ and PAR_amb_ values.

To assess what controlled the switch between Models 2 and 3, we performed GMM (using qL_high-amb_, P^+^_high-amb_, T_leaf_ as inputs). Four distinct clusters were observed (see symbol colours, figure 8*a*). Intercluster comparisons show that points in clusters 1 and 2 fell exclusively in the region predicted for Model 3. Cluster 3 fell entirely within the region predicted for Model 2. Cluster 4 extended between these regions, possibly indicating contributions from both mechanisms. The clusters falling in the Model 3 region were associated with relatively low T_leaf_ (figure 8*c*) and PAR_amb_ (figure 8*d*), compared with those associated with Model 2 or intermediate behaviours, suggesting that Model 2 prevailed at higher T_leaf_ and/or PAR_amb_, while Model 3 prevailed at lower values. Within the GMM clusters (electronic supplementary material, figure S13), qL_high-amb_ was dependent predominantly on T_leaf_ (cluster 3), PAR_amb_ (cluster 1), or both (clusters 2 and 4). This dependence suggests that T_leaf_ and PAR_amb_ acted either independently or cooperatively, depending on conditions, affecting the propensity for photosynthesis to adopt Model 2 or 3 behaviours. As a first-order test of the robustness of these clusters by re-analysing randomly selected subpopulations of the data. As discussed in the legend to electronic supplementary material, figure S14, we obtained comparable results, i.e. that we would interpret in similar ways, with as few subpopulations as small as 25% of the full dataset, suggesting that the clustering approach was reasonably robust.

The data in figure 8 show that, at lower T_leaf_ and PAR_amb_, qE activation was suppressed despite light-induced increases in *pmf*, and that this behaviour was associated with accumulation of electrons on Q_A_ but oxidation of P_700_ (figure 8*a*), suggesting that, under these conditions, light-induced increases in ΔpH caused slowing of the cytochrome *b_6_f* complex (PCON), but that the qE response lagged behind or was completely suppressed, leading to Model 3 behaviour. It is known that, initially after an abrupt increase in PAR, increased thylakoid *pmf* is stored as Δ*ψ*; conversion of Δ*ψ* to ΔpH is controlled by the movement of counterions across the thylakoid membrane, and protonation of lumenal proton buffering groups occurs over the seconds to tens of seconds time scale [25,61–63], and is dependent on the activities of various thylakoid ion transporters [9–11]. However, little is known about the natural diversity of Δ*ψ*/ΔpH balancing.

It has been shown that the lumen pH-dependencies of qE and PQH_2_ oxidation by the cytochrome *b_6_f* complex are tightly coordinated, so that increased lumen acidity activates photoprotection prior to PCON, presumably to prevent the accumulation of reduced Q_A_ [15]. However, these experiments were performed under more slowly changing (near steady-state) conditions in the laboratory, and our results suggest that this coordination can be defeated under real-world conditions in the field, especially when T_leaf_ is low and PAR fluctuates rapidly. This discoordination can have strong implications for photodamage, as it has been shown that high thylakoid *pmf* can greatly accelerate PSII recombination reactions, especially when Q_A_ is reduced, leading to ^1^O_2_ production [28,29,32]. It thus seems reasonable to suggest that the shift from qE to PCON at low T_leaf_ will increase the rates of photodamage.

There are several possible mechanisms by which the response of qE can be uncoupled from increased *pmf*. Longer-term dependencies of NPQ on temperature have been reported under both field [64–66] and laboratory [67,68] conditions. The current work shows effects on rapid NPQ and LEF changes, which can be related to distinct mechanistic models. For example, it is known that the xanthophyll cycle is strongly temperature dependent, though the general observation is that zeaxanthin tends to accumulate at lower temperatures due to a slowing of the epoxidation of zeaxanthin [67–69]. Interestingly, we would expect the accumulation of zeaxanthin to augment, rather than suppress qE responses as we have observed in the current results. Lumen acidification may also be rate limiting for formation of qE. While rapid increase in light can result in nearly instantaneous increases in Δ*ψ*, formation of ΔpH and lumen acidification require counterion transport processes, which tend to be slow, and thus lumen acidification lags behind [25,29], and it is possible that this process is substantially slowed at low temperature. Other possible limitations include temperature-dependence of conformational rearrangement of antenna complexes following protonation of PsbS [70,71], which in turn may be related to the interactions among thylakoid proteins, lipids and ultrastructure [12,45,72,73]. The current data do not allow us to discriminate between these models, but the work suggests conditions and species under which such limitations occur, and how they may impact plant productivity or resilience.

## 5. Conclusion: current limitations and prospects for open science-led efforts to understand and improve photosynthesis

There are intense, ongoing efforts to improve photosynthesis, yet the importance of the responses of photosynthesis under fluctuating, real-world conditions are just now being recognized. In particular, we lack understanding of the extents and impacts of these responses, as well as their mechanisms and genomic control, which will be critical to achieving field-relevant improvements in efficiency and robustness, especially in a changing environment.

Here, we demonstrate methods and tools to assess the light responses of photosynthetic processes under real-world conditions, and use them to explore the factors that limit the capacity of plants to use or dissipate rapidly increased PAR. A major outcome is that, despite the complexities of field environments, clear behavioural patterns can be resolved, as long as the experiment contains a sufficient number of points taken over a large environmental space, and that includes environmental metadata. Such combinations of rapid measurements allowed us to test for various models over broad scales by looking for internally consistent relationships among the various measured parameters. For example, we observed no evidence for Model 1 (limitation at the acceptor side of PSI) behaviour in the current study, but we do not exclude the possibility in different species and/or different environmental conditions. The analysis supports the operation of Model 2, the rapid activation of NPQ resulting in net oxidation of Q_A_^−^ and Model 3, the strong activation of PCON, resulting in accumulation of Q_A_^−^. We surmised that Model 2 behaviour would be the most photoprotective, while Model 3 type behaviour would probably lead to photodamage, though we do not have independent endpoint measurements (e.g. yield, growth rates, etc.) to validate that the propensity for Model 3 behaviour has long-term consequences. Further, the models are not exclusive, and there will almost certainly be cases, e.g. cluster 4 in figure 8, where intermediate behaviours will be apparent, either because of co-limitations among multiple processes or heterogeneity between chloroplasts in the leaf samples.

We also emphasize that the data presented here were intended to introduce the approaches and methods, and thus leave a number of questions unanswered, but set up the approach to further study. The origins of these effects may include several classes of processes [31,74] that may differ under different conditions [75], including induction of downstream assimilatory reactions and metabolic pools [76,77], downstream sink reactions [78], redox regulation [79,80], balancing between the production and consumption of ATP and NADPH [1,49], ion homeostasis and regulation of thylakoid *pmf* [25,81], low stomatal aperture that may lead to transient depletion of internal CO_2_ levels. Distinguishing these will probably require more detailed phenotyping and biochemical [10,60,82] modelling [31] and genomics and genetics approaches [83].

The accessibility of the tools should allow larger numbers of researchers to answer these types of questions over a broader set of results. This approach was made possible by the combination of several open science advances. Collation of large amounts of data and metadata through the MultispeQ and PhotosynQ platforms [34], allowed us to explore the interdependencies of multiple responses and environmental conditions (metadata). The GMM methods allowed us to explore the interactions among multiple environmental parameters and photosynthetic responses, and test for the participation of distinct mechanistic models to explain the limitations to photosynthesis under field conditions, leading to the identification of distinct limitations in the rapid activation of NPQ and LEF at low temperature. Finally, making all tools, protocols and analytical methods available in directly usable forms, the project can be readily expanded to include multiple environments and species, as well as alternative models.

## Data accessibility

Primary data are available on the photosynq.org site under the project ‘rapid-ps-responses-pam-ecst-npqt-mint-dmk’. Data cleaning and analysis code is available in a GitHub repository (https://github.com/protonzilla/Light-Potentials-in-Field).

The data are provided in electronic supplementary material [84].
